# Glucocorticoids and Trabecular Bone Score

**DOI:** 10.25122/jml-2019-0131

**Published:** 2020

**Authors:** Florica Sandru, Mara Carsote, Mihai Cristian Dumitrascu, Simona Elena Albu, Ana Valea

**Affiliations:** 1. Department of Dermatology, Elias Emergency University Hospital, Bucharest, Romania; 2. “Carol Davila” University of Medicine and Pharmacy, Bucharest, Romania; 3. Department of Endocrinology, C.I.Parhon National Institute of Endocrinology, Bucharest, Romania; 4. Department of Gynecology, Emergency University Hospital, Bucharest, Romania; 5. Department of Endocrinology, Clinical County Hospital, Cluj-Napoca, Romania; 6. “Iuliu Hatieganu” University of Medicine and Pharmacy, Cluj-Napoca, Romania

**Keywords:** Glucocorticoid, cortisol, trabecular bone score

## Abstract

TBS (Trabecular Bone Score) is the latest tool for clinicians to evaluate bone micro-architecture based on a pixel greyscale, which is provided by lumbar dual-energy X-ray absorptiometry (DXA). Its use enhances fracture prediction in addition to DXA-BMD (Bone Mineral Density). This is independent of fracture risk assessment (FRAX) and DXA results. We present a narrative review regarding the connection between TBS and Glucocorticoids (GC), either as a drug used for different conditions or as a tumor-produced endogenous excess.

TBS is a better discriminator for GC-induced vertebral fractures compared to DXA-BMD. This aspect is similarly available for patients with osteoporosis diagnosed by DXA. TBS is inversely correlated with the cumulative dose of GC (systemic or inhaled), with disease duration, and positively correlated with respiratory function in patients with asthma. Low TBS values are found in females with a T-score at the hip within the osteoporosis range, with diabetes mellitus, or who use GC. Lumbar TBS is a screening tool in menopausal women with type 2 diabetes mellitus. TBS is an independent parameter that provides information regarding skeleton deterioration in diabetic patients receiving GC therapy in a manner complementary to DXA-BMD. TBS might become an essential step regarding the adrenalectomy decision in patients with adrenal incidentaloma in whom autonomous cortisol secretion might damage bone micro-architecture. TBS currently represents a standard tool of fracture risk evaluation in patients receiving GC therapy or with endogenous Cushing’s syndrome, a tool easy to be applied by different practitioners since GCs are largely used

## Introduction

TBS (Trabecular Bone Score) is the latest tool for clinicians to evaluate bone micro-architecture based on a pixel greyscale, which is provided by lumbar dual-energy X-ray absorptiometry (DXA) acquisition [1, 2]. Thus, bone quality data are provided, and it becomes important for different conditions, including prolonged glucocorticoid exposure of any origin, exogenous, or endogenous [1-3]. The use of TBS, in addition to DXA-BMD (Bone Mineral Density), enhances the fracture prediction [2, 3]. The fracture prediction is independent of the fracture risk assessment (FRAX) or DXA [1-3]. Some subgroups of patients with secondary osteoporosis, including GC-related osteoporosis, are advantaged by TBS use [2-4].

Our aim is to present a literature review regarding the connection between TBS and glucocorticoids either as a drug used for different conditions or as a tumor-produced endogenous excess.

## Material and Methods

This is a narrative review of the literature. The tool of research was mainly the PubMed database. A selection of 50 papers has been made based on clinical relevance through a multidisciplinary approach.

## Results

### Trabecular Bone Score

Since 70% of osteoporotic fragility fractures occur in subjects with DXA-BMD according to osteopenia ranges, there is a need for tools improvement, tools that clinicians of different specialties must have in order to assess the fracture risk [5]. TBS has gained importance during the last six years in daily practice in addition to traditional DXA, which remains the gold standard [6]. Moreover, TBS data may be combined with FRAX [7]. Apart from primary osteoporosis, secondary types like those seen in primary hyperparathyroidism, type 2 diabetes mellitus, and GC-induced are special areas of TBS applications [8-10].

### Glucocorticoids and skeleton

GC-related osteoporosis involves either the GC as medication or endogenous production of cortisol due to a Cushing’s syndrome of an adrenal or a pituitary cause [11, 12]. It is considered the most frequent type of secondary osteoporosis with a consequent medical and economic severe burden, while the exogenous (also named iatrogenic) Cushing’s syndrome is the most common type of Cushing’s syndrome [13, 14]. An additional cause of bone loss, as seen in menopause-related estrogen deficiency, increases TBS and BMD deterioration [15]. Also, a baseline inflammatory status or the primary condition that indicated GC is a direct contributor to bone loss [16]. The underlying mechanisms of GC-induced osteoporosis include autophagy of bone cells, the shift between osteoblastogenesis and adipogenesis, high bone resorption, and low bone formation [17-20]. The fracture risk is highest during the first 6 months of GC therapy, and then it decreases depending on the dose [19].

### The use of glucocorticoids and TBS

The use of GC is widely accepted, and long-term complications are expected. The changes of TBS are revealed based on different studies as a new reflection of bone quality anomalies and their dynamics. TBS provides insights into micro-architecture, an aspect that could bring useful information for any clinician that needs to prescribe GC.

In 2019, Florez H. *et al*. published one study on 127 subjects (63% were women with an average age of 62 years) treated with GC for different autoimmune diseases for a mean of 47.7 months that developed osteoporosis [21]. TBS was more affected than BMD-DXA [21]. 17% of them had prevalent vertebral fractures, and overall, 28% of them had any type of fractures while the DXA T-score established the diagnosis of osteoporosis in 29% of cases; 52% of them had a TBS of less than 1.230 that showed deteriorated micro-architecture [21]. This value was found more often in the subgroup with osteoporosis diagnosed by DXA (76% versus 38%) or with prevalent fractures (69% versus 36%) [21]. A better specificity is obtained when BMD is added to TBS (a 0.89 increase for the vertebral fracture group and 0.9 increase for the group with any type of fracture) [21]. TBS is a better discriminator for GC-induced vertebral fractures compared to DXA-BMD [21].

Another controlled study on 265 menopausal women receiving GCs showed that TBS is statistically significantly lower in patients with polymyalgia rheumatic than the subjects with rheumatoid arthritis or control (an average of 1.317 versus 1.336 versus 1.373, respectively), and it becomes a better discriminator for the patients with prevalent vertebral fractures and menopausal osteoporosis [22]. This tool also provides a valuable discriminatory power for osteoporotic fragility fractures in menopausal females with a prior diagnosis of rheumatoid arthritis, and a relationship with the cumulative dose of GC is also found [23].

In 627 patients with asthma treated with GC, one controlled cohort study showed lower values of TBS compared to non-asthma subjects (1.320 versus 1.360, respectively; p-value = 0.001) while lumbar DXA-BMD was not different between the mentioned groups [24]. TBS is inversely correlated with the cumulative dose of GC (systemic or inhaled), and with disease duration, and positively correlated with the respiratory function [24]. Thus, TBS provides an early hallmark of bone deterioration under GC therapy on persons who are candidates for long-term GC exposure [24].

Short-term of high-dose GC used for Graves’ orbitopathy showed no changes of TBS or BMD but showed transient suppression of bone resorption markers, so it seems that bone micro-architecture is not affected if methylprednisolone is used for three months [25].

Extra encouraging data are provided by the low-dose GC protocol after kidney transplant [26]. One prospective cohort study in patients who did not receive specific drugs for bone protection revealed that 12 months after surgery, lumbar BMD has a small decrease of 2.1% from baseline, but this was not available for BMD at other sites or TBS [26].

37, 176 subjects enrolled in the Manitoba Registry showed lower TBS values in females with a T-score at the hip within the osteoporosis range, with diabetes, or who use GC [27]. Clear reclassification improvement is seen in cases with high GC regimes (a percent of 3.9% for major osteoporotic fractures) [27].

The FRODOS cohort that included 2,257 menopausal women showed that decreased TBS is correlated with age, DXA T-score, weight, GC therapy, type 2 diabetes mellitus, and prevalent osteoporotic fractures [28]. The cohort had a mean TBS of 1,203, and 55% of subjects had the lowest TBS grade (≤1,230), which is registered as degraded micro-architecture [28].

A pre-post controlled study also showed that FRAX adjusted for TBS is statistically significantly higher on patients with major osteoporotic fractures receiving GC therapy [29].

### Endogenous Cushing’s syndrome and TBS

Both Cushing’s disease and adrenal Cushing’s syndrome have been found at risk for low TBS [30, 31]. A cohort of 182 patients with endogenous Cushing’s syndrome showed that the best predictor of fragility fractures is the 24-hour urinary free cortisol [30]. The subjects with corticotropinomas had a mean TBS of 1.207, and low values are detected even in young patients. Overall, it seems that the disease activity is a more powerful predictor than TBS [30].

One retrospective study that included 110 patients with endogenous Cushing’s syndrome showed that 24% of patients have a BMD-based diagnosis of osteoporosis, and 43% had degraded TBS with lower TBS in a clinically apparent disease versus non-secretor incidentaloma at the level of the adrenal glands [31].

After the surgical approach of the tumor that is causing Cushing’s syndrome, TBS improves faster than BMD [31]. Some data reported an accelerated bone repair after the control of Cushing’s disease, and TBS might completely normalize [32]. Thus, TBS becomes a part of the standard evaluation protocol in endogenous Cushing’s syndrome [33]. Moreover, in subjects with Cushing’s syndrome, as in normal, TBS correlates with BMD and serum osteocalcin [34]. Also, it represents a good discriminator for vertebral fractures [35]. Finally, TBS became part of the standard osteoporosis assessment [36].

## Discussion

We choose to discuss some topics which do not yet have a clear answer.

### Adrenal incidentaloma

TBS seems a useful resource for detecting the patients at risk for fractures related to autonomous cortisol secretion, which is found in almost half of the adrenal incidentalomas [37, 38]. TBS might become a step in adrenalectomy decisions in these cases [39]. Subclinical Cushing’s syndrome also includes a subtle bone loss, which is more evident in menopausal women or in cases with long-term exposure [40]. This subclinical GC exposure is associated with a 2.2% TBS reduction, opposite to clear non-secretor incidentalomas [41]. TBS inversely correlates in these patients with serum cortisol after the dexamethasone suppression test [41]. Yet, TBS is not included in the guidelines of adrenal incidentalomas.

### Confounding factors in assessing the effects of the glucocorticoid

Many patients developing Cushing’s syndrome because of GC excess also have diabetes mellitus, obesity, or hyperlipemia [42-44]. These complications may cause a deterioration of TBS, which is additive to the GC effect on the micro-architecture [42]. Lumbar TBS is a screening tool in menopausal women with type 2 diabetes mellitus [44]. TBS is an independent parameter that provides information regarding skeleton deterioration in diabetic patients under GC therapy in a manner complementary to DXA-BMD [45]. There is still a matter of debate regarding which is the exact change of TBS correlated with diabetes, with GCs or with both [46].

### Anti-osteoporotic therapy for glucocorticoid osteoporosis: TBS values

There are still limited data regarding the magnitude of TBS changes in patients on GCs and anti-osteoporotic drugs [47]. It seems that changes in lumbar BMD are more evident than those of lumbar TBS in cases treated with specific medication for osteoporosis [47]. The changes of TBS in these cases only partially reflect the consecutive reduction of the fracture risk [47]. Subjects with GC therapy who were treated with oral alendronate or teriparatide had a significant increase of lumbar BMD, but TBS increases only after osteoanabolic medication [48]. Since GC- induced osteoporosis has a pronounced component of reduced bone formation, TBS might be a good detector of micro-architecture changes under bone-forming agents in this type of secondary osteoporosis [48]. Also, TBS may bring important information in individuals at risk for fractures as it happens on GCs exposure that is close to the interventional threshold of therapy and an additional tool as bone micro-architecture deterioration may be crucial for anti-osteoporotic drug decision [49]. The International Society for Clinical Densitometry (ISCD) concluded in 2019 that TBS is not clinically relevant for the follow-up of patients taking bisphosphonates or denosumab. However, there is no clear data up to now, while the good TBS response at teriparatide or abaloparatide is more clinically relevant [50].

## Conclusion

TBS currently represents a standard tool for fracture risk evaluation in patients receiving GC therapy or patients with endogenous Cushing’s syndrome. It is an easy tool to apply by different practitioners since GCs are largely used.

## Conflict of Interest

The authors declare that there is no conflict of interest.
